# How patients experience respect in healthcare: findings from a qualitative study among multicultural women living with HIV

**DOI:** 10.1186/s12910-024-01015-1

**Published:** 2024-03-27

**Authors:** Sofia B. Fernandez, Alya Ahmad, Mary Catherine Beach, Melissa K. Ward, Michele Jean-Gilles, Gladys Ibañez, Robert Ladner, Mary Jo Trepka

**Affiliations:** 1 School of Social Work, Robert Stempel College of Public Health and Social Work, Florida International University, 11200 SW 8th St, Miami, FL 33199 USA; 2 Research Center in Minority Institutions, Florida International University, 11200 SW 8th St, Miami, FL 33199 USA; 3 Department of Medicine, Division of General Internal Medicine, Johns Hopkins University, 2024 East Monument Street, Baltimore, MD 21205 USA; 4 Department of Epidemiology, Robert Stempel College of Public Health and Social Work, Florida International University, 11200 SW 8th St, Miami, FL 33199 USA; 5 Department of Health Promotion and Disease Prevention, Robert Stempel College of Public Health and Social Work, Florida International University, 11200 SW 8th St, Miami, FL 33199 USA; 6 Behavioral Science Research Corporation, 2121 Ponce de Leon Boulevard, Coral Gables, FL 33134 USA

**Keywords:** Patient-centered care, Bioethics, Respect, HIV care and treatment

## Abstract

**Background:**

Respect is essential to providing high quality healthcare, particularly for groups that are historically marginalized and stigmatized. While ethical principles taught to health professionals focus on patient autonomy as the object of respect for persons, limited studies explore patients’ views of respect. The purpose of this study was to explore the perspectives of a multiculturally diverse group of low-income women living with HIV (WLH) regarding their experience of respect from their medical physicians.

**Methods:**

We analyzed 57 semi-structured interviews conducted at HIV case management sites in South Florida as part of a larger qualitative study that explored practices facilitating retention and adherence in care. Women were eligible to participate if they identified as African American (*n* = 28), Hispanic/Latina (*n* = 22), or Haitian (*n* = 7). They were asked to describe instances when they were treated with respect by their medical physicians. Interviews were conducted by a fluent research interviewer in either English, Spanish, or Haitian Creole, depending on participant’s language preference. Transcripts were translated, back-translated and reviewed in entirety for any statements or comments about “respect.” After independent coding by 3 investigators, we used a consensual thematic analysis approach to determine themes.

**Results:**

Results from this study grouped into two overarching classifications: respect manifested in physicians’ orientation towards the patient (i.e., interpersonal behaviors in interactions) and respect in medical professionalism (i.e., clinic procedures and practices). Four main themes emerged regarding respect in provider’s orientation towards the patient: being treated as a person, treated as an equal, treated without blame or prejudice, and treated with concern/emotional support. Two main themes emerged regarding respect as evidenced in medical professionalism: physician availability and considerations of privacy.

**Conclusions:**

Findings suggest a more robust conception of what ‘respect for persons’ entails in medical ethics for a diverse group of low-income women living with HIV. Findings have implications for broadening areas of focus of future bioethics education, training, and research to include components of interpersonal relationship development, communication, and clinic procedures. We suggest these areas of training may increase respectful medical care experiences and potentially serve to influence persistent and known social and structural determinants of health through provider interactions and health care delivery.

**Supplementary Information:**

The online version contains supplementary material available at 10.1186/s12910-024-01015-1.

## Background

For the past nearly half century, the ethical education of health professionals has been dominated by Beauchamp and Childress’s Principles of Biomedical Ethics, first published in 1979 and now on its eighth edition [1–4]. The principles described in this text include respect for patient autonomy, beneficence, nonmaleficence, and justice, and, by far, the principle that received most attention has been ‘respect for autonomy.’ Because respect for autonomy is also referred to as ‘respect for persons,’ and because – regardless of what it is called – discussions of respect focus exclusively on patient autonomy, health professionals explicitly learn that the obligation to respect persons primarily entails informed consent, noninterference with patient choice, and honesty. Although the use of these principles as a moral guide in clinical medicine has been challenged [5, 6], the principles remain staunchly defended [7, 8].

As important as respecting patient autonomy is, it does not fully describe aspects of what it means, from patients’ perspectives, to be treated with respect [9–11]. Patient experiences of respect, and disrespect, are primarily interpersonal, and therefore patient accounts of respect tend to focus on dignity more so than autonomy [12]. From patients’ perspectives, instances of disrespect may include being treated rudely, ignored, or looked down on [13]. On the other hand, instances of respect, from patients’ perspectives, may include being known as a person, treated kindly, or simply being acknowledged by clinicians [9–11]. There is considerable literature on behaviors we tend to associate with respect, specifically literature that looks at important aspects of effective interpersonal communication. For example, there is research on how aspects such as shared decision making, communicating empathetically, cultural sensitivity, attentive listening, and building rapport/trust are critical for effective physician and patient interactions [14–19]. Indeed, the Institute of Medicine defines patient-centered care as care that includes compassion, empathy, and is respectful of patient’s values, preferences, and needs [20]. Yet, while respect is widely known and accepted as essential to physician/ patient relationships, it is rarely defined and our understanding of how patients perceive respect is limited. Moreover, interpersonal behaviors, which are fundamental to how patients experience respect, have been largely relegated to a different sphere of medicine – the world of teaching interpersonal communication skills to clinicians – and away from teaching the ethical responsibility for respect of persons in medicine. The adverse consequence on the culture of healthcare is that clinicians may not feel morally obligated to respect the dignity of patients or to treat them with interpersonal respect.

Although previous work exploring the meaning of respect in different patient groups has found some consistent themes (e.g., being treated as a person), there have also been some important differences related to the particular background and context of each patient group. This highlights the need to look at multiple perspectives. Therefore, the aim of this study was to explore the meaning and significance of respect for a group of racially and ethnically diverse, low-income women living with HIV (WLH), in an attempt to offer a more robust conception of what ‘respect for persons’ entails in medical ethics.

## Methods

### Study design and setting

We conducted an analysis of qualitative data that was collected from a sample of racially and ethnically diverse, low-income women receiving HIV care from the Ryan White Program (RWP) in Miami-Dade County, Florida. Semi-structured, in-depth interviews were conducted September 2019–March 2020 as part of a larger study that aimed to explore women-centered practices that facilitate retention in care and adherence medication among WLH [21]. Women were eligible to participate if they: (a) were enrolled in the RWP for at least 6 months; (b) were at least 18 years of age; (c) identified as Hispanic/Latina, Haitian, or Black/African American; (d) spoke English, Spanish, or Haitian Creole, and (e) had previously given consent to be contacted for RWP research. To ensure inclusion of women from various backgrounds, we recruited women from each of the three prominent racial and ethnic minority groups in Miami-Dade County (i.e., Hispanic/Latina; Haitian; and Non-Hispanic, Non-Haitian, Black/African American women). Women were informed about the research study via telephone and recruitment flyers. If interested, research staff then arranged to meet at local RWP medical case management sites where they further reviewed the purpose of the study and informed consent. If women were still interested and provided written informed consent, the interview was then conducted in a private room within the medical case management site.

### Data collection and procedures

Interviews were conducted by a team of 6 interviewers using a semi-structured interview guide that was developed by members of the research team with experience in culturally sensitive data collection among people living with HIV. Interview guides were designed as a part of the larger parent study and aimed to explore women’s perspectives on patient-centered care using the Institute of Medicine’s (IOM) definition of patient-centered care, barriers to and facilitators of care retention and adherence to medication, as well as asked specific probing questions to illuminate instances when women felt they were treated with respect during interactions with their HIV providers and/or nurses. The complete interview guide used for the parent study is included in this manuscript (see Additional file 1).

Interviews lasted about 1-hour and conducted in English, Spanish, or Haitian Creole, depending on the participant’s language preference, by an interviewer who was fluent in the preferred language. After interview completion, digital audio recordings were transcribed verbatim and de-identified for subsequent analysis. Steps to ensure the accuracy of the transcript data were taken including random linguistic and conceptual equivalence checks between the original language transcripts and the translated transcripts. Participants received a $60 gift card as compensation for study participation. All study procedures were approved by the Social Behavioral Institutional Review Board of Florida International University.

### Analysis

For this study, we focused our analysis on a subgroup of interviews from the parent study that were explicitly asked one particular prompt question: “What are examples of times you felt you were treated with respect by your doctor and nurse?”. Transcript responses from all of the parent study interviews (*n* = 74) were reviewed in entirety by one of the primary researchers; all responses to this question were extracted for analysis, resulting in a total of 57 participant responses for inclusion in the current analysis. A thematic analysis approach was used to determine themes. In line with a traditional thematic analysis method as outlined by Braun and Clarke, the researchers followed the following process for conducting the analysis [22]. First, all coders read through the data to familiarize themselves with the data set. Then, one primary researcher went through the data and created initial codes for responses. Next, two other researchers coded the data, independently, using initial codes as a guide. After independent coding, the three researchers met over several analytic meetings to determine final definitions of the codes, discuss agreement of code assignments, identify emerging codes, merge codes into larger themes, and select representative quotes [22]. Because participants often touched on more than one theme within one statement, we present full quotes below, underlining the specific part of the quote that pertains to the corresponding theme. Trails of the coding process were kept to ensure reliability and transparency of coding and to aid the researchers in the process of transforming codes into larger thematic categories [23]. We used Excel as an organizational and data management tool.

It is important to note key characteristics of the researchers involved in this study. The interviewers were hired by Behavioral Science Research Corporation, the evaluator for the Ryan White Program in Miami-Dade. Hired interviewers received training from the research team on how to conduct culturally sensitive interviews in order to increase the comfort level of participants and build rapport necessary for authentic data collection. The interviewers were not case managers and were not involved in the care of participants. The researchers who conducted the transcriptions were not those who conducted the interviews. These researchers were also trained by the research staff on how to transcribe data verbatim. Transcribers were fluent in the language that the interview was conducted, as well as fluent in English to ensure language equivalency of translated transcriptions. The data analysis team (3) was a multicultural group of women. It is important to note the positionality of the researchers who conducted this analysis. The women were based in Florida and Maryland with professional backgrounds in medicine, bioethics, and social work and came from a range of career levels (i.e., research assistant, assistant professor, and associate professor). Two of the analysts were part of the parent project research team and provided initial input in the development of the interview protocol. Neither of these analysts was a part of data collection. One analyst was not involved in the parent project during project design or data collection. The data analysts met several times to discuss assignment of codes and consensus on the creation of themes.

## Results

### Participant characteristics

Data from a total of 57 women was in included in the analysis. The majority of women identified racially as Black (66.7%); this included participants across different ethnic backgrounds. Women identified as Hispanic/Latina (38.6%), Non-Hispanic Black/African American (49.1%), and Haitian (12.3%). Among participants, 7.0% were 18–35 years old, 28.1% were 36–49 years old, 38.6% were 50–59 years old and 26.3% were 60 years of age or older. The interviews were conducted primarily in English (61.4%), followed by Spanish (29.8%), and Haitian Creole (8.8%). See Table 1 for further demographic and background characteristics of the sample.

**Table 1:** Participant Sociodemographic Characteristics (*N* = 57)

Characteristics	*n* (%)
**Gender**	
Women	57 (100.0)
**Racial background**	
Black/African American	38 (66.7)
White	19 (33.3)
**Ethnic background**	
Hispanic/Latina	22 (38.6)
Non-Hispanic, non-Haitian, Black/ African American	28 (49.1)
Haitian	7 (12.3)
**Age group (years)**	
18–35	4 (7.0)
36–49	16 (28.1)
50–59	22 (38.6)
60+	15 (26.3)
**Education**	
Less than high school	19 (33.3)
High school	19 (33.3)
Some higher education	19 (33.3)
**Country of birth**	
US	18 (31.6)
Haiti	6 (10.5)
Honduras	9 (15.8)
Jamaica	5 (8.8)
Bahamas	5 (8.8)
Cuba	3 (5.2)
Nicaragua	2 (3.5)
Venezuela	2 (3.5)
Turks/Caicos	2 (3.5)
Puerto Rico	2 (3.5)
Chile	1 (1.8)
Dominican Republic	1 (1.8)
St. Thomas	1 (1.8)
**Language of interview**	
English	35 (61.4)
Spanish	17 (29.8)
Haitian Creole	5 (8.8)
**Years living in Miami**	
0–9	5 (8.8)
10–19	14 (24.5)
> 20	38 (66.7)
**Marital Status**	
Single	31 (54.4)
Married/cohabiting	10 (17.5)
Widow	10 (17.5)
Separated/divorced	6 (10.5)

### Themes

Participants described respect in a number of ways, and often blended different aspects of respect into a single response, highlighting the multidimensional experience of respect within an individual. Thematic results are grouped into two overarching classifications: respect that manifested in the physicians’ orientation towards the patient and respect in aspects related to medical professionalism. We did so because the implications for each can be conceptualized in distinct ways. Physicians’ orientation towards the patient includes interpersonal aspects and behavioral examples demonstrated during patient/physician interactions. Respect in medical professionalism includes clinic procedures and has implications for clinic scheduling and organizational practices. These themes, definitions, and examples are summarized in Table 2.

**Table 2:** Respect Themes and Corresponding Definitions, Example Descriptions, and Illustrative Quotes

Theme	Definition	Example Descriptions	Illustrative Examples from Direct Quotes
*Respect in provider’s interpersonal orientation towards patient*			
Treated as a person	To be known as a person; inquiring about personhood; To be more than a patient	Using patient’s name to greet them; Looking at patient in the eye; Asking patient how they are doing; Asking about non-medical aspects of an individual (e.g., work, family, friends) Utilizing manners that show friendliness including smiles, hugs, and greetings	“When I first come in through the door she says, ‘Good morning, (Name),’ with a smile on her face. That’s respect right there, you know. That woman could we working the whole day, she don’t have something slick on her mouth. Had a rough day, you know. She make me come in, she smile, she always tells me “good morning (Name), how you doing?” (55 years old, Black, Non-Hispanic, from Turks and Caicos). “Before we start talking about the exams or labs, he always asks me how I am, work, and the family. There is always a little conversation before beginning discussing health issues, always” (41 years old, White, Hispanic, from Nicaragua). “When he come in like make me check in, and he see me he say, ‘Hey, (Name).’ He give me a little hug. You feel so welcomed. Everybody hug me, smiling, have me a little gift… As we were friend, and that’s where we take it with friendship. Oh (name) take care of yourself, when he leaving he give me a hug. He not like they better than us, we all want people but you could feel as a doctor.” (51 years old, Black, Non-Hispanic, from Jamaica).
Treated as an equal	To be collaborators (physician and patient) in care and treatment decisions; To communicate in a way that recognizes the patient’s capacity for understanding and the patient’s value in health decision making	Allowing and encouraging patients to provide input/ ask questions; Providing opportunity for listening/ explaining; Demonstrating partnership with client; believing the patient’s experience	“Any kind of questions I would ask her, again she wouldn’t look down on me, if I needed anything, she would provide it. If she couldn’t, she would refer me to someone that could” (44 years old, White, Hispanic, from the US). “But over the years, I’ve been with those same people. And like, I say, anything I need, any questions I have concerning my health, like my medications. For instance, I might be on this type of medication, they felt the need to change it for whatever reason, I can, I talk to them and they’ll tell me why they think this one is better to me opposed to the other one I was taking. You know, taking this stuff like that” (67 years old, Black, Non-Hispanic, from Jamaica).
Treated without blame/ without prejudice	To avoid putting shame or guilt on the patient; To act without prejudice or stigma towards the patient	Standing up for the patient; Avoiding talking down to a patient; Avoiding placing blame on patient; Treating without prejudice	“From the first time that I got there, they sent me there with that doctor, and he made me come in to the office and treated me respectfully. ‘What’s wrong with you? How are you? How did such a thing happen to you?,’ and respectfully. When my husband would yell at me, he would say, ‘Stop, stop, she’s not here for you to yell at her.’” (70 years old, White, Hispanic, from Honduras). “He never speaks badly, angry, though sometimes I’m late for the appointment and the doctor seeing me starts laughing as saying “again” but always been very respectful” (41 years old, White, Hispanic, from Nicaragua).
Treated with concern/ Emotional support	To show warmth, love, and to provide comfort to the patient; To provide support when the patient expresses emotions; Expressing concern To show interest and concern for the patient’s health	Acknowledging, consoling and comforting the client in times of distress; Providing warmth and love towards patient; Expressing interest and concern for the patient; Providing reassurance	“He listens to me and as, like I said, as I had different experiences during the years, he watched me grow and became a flower. And he would say that too because when I came to him, I was like afraid and everything, you know, of course, being first diagnosed, you think you’re going to die tomorrow. And, you know, they helped me through it. You know what I mean. Before –years later. Like just like a year ago now, I finally used the, what is it called, the mental health service? Yeah, but I didn’t need that because my whole team was like talking to me, consoling me. They supported me, like physically and emotionally. And some prayed with me. So like, it I can… I really have a good team” (41 years old, Black, Non-Hispanic, from The Bahamas). “I remember I had got really sick. I had an abscess right under my left booty check, and I was, I don’t care who you are, I don’t want nobody looking at me. She was like, ‘(Name), I got to look.’ I was like, ‘No doc, I’m just gonna tell you about it.’ But she made me start laughing and got me to such a comfortable space where like I’m okay with her looking in certain parts, because she made me feel like baby. ‘I’m just trying to help you. Don’t feel embarrassed.’ Because you know, I’m like 240 [pounds]… So yeah, we’re there now” (51 years old, Black, Non-Hispanic, from the US).
*Respect in medical professionalism*			
Availability	To have timely access to the physician	Allowing the patient to speak to and see the doctor without appointments; making an accommodating schedule for the patient	“Most doctors, you have to have appointments. My doctors not like that. I would walk in there right now and he’d see me, if he has 10. If he can’t get to what I’m there for then he will schedule but if I walk in my doctor’s office, my care provider, they will see me. It’s never that you have to have an appointment to see your doctor. That’s why I like him” (45 years old, Black, Non-Hispanic, from the US). “If I go in to talk to him, even if I don’t have an appointment I can go in as a walk-in, and I get to see him right away, you know. It’s everything” (64 years old, Black, Non-Hispanic, from The Bahamas).
Privacy	To abide by confidentiality and consent	Asking consent when others are in the physician room; Providing spaces and utilizing practices that allow for privacy in waiting rooms	Negative case: “Your patient already sign in and are put a waiting room, back there for your patients to sit in. Patients don’t wanna be sitting in that open environment” (58 years old, Black, Non-Hispanic, from the US). Negative case: “There is always someone there, it’s disrespectful. I would want more privacy. No more students… For example, last time I was in a room… and there was no privacy… It was a new doctor, and everyone noticed I was HIV. Because she screamed it like, ‘Oh bring me the chart because she is HIV!’” (47 years old, White, Hispanic, from Honduras).

**Fig. 1:**
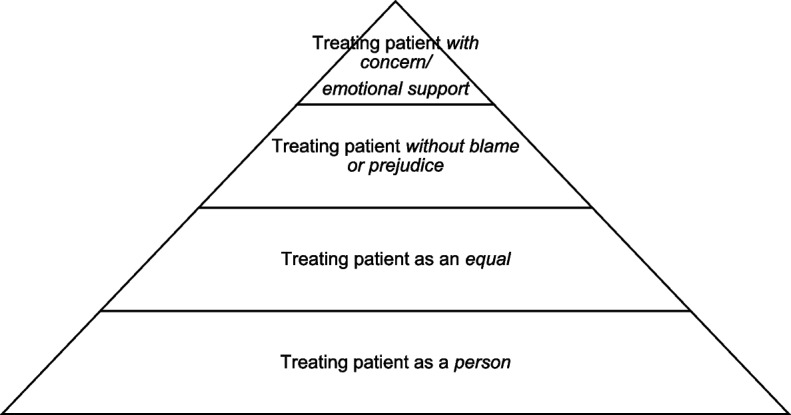
Themes of Respect in Providers’ Orientation Towards their Patients

Four main themes emerged regarding respect in provider’s orientation towards the patient, being treated: (1) as a person, (2) as an equal, (3) without blame or prejudice, and (4) with concern/emotional support. It is important to note that these four themes overlap and are not mutually exclusive categories. We present them in a pyramid (see Fig. 1) to demonstrate interconnectedness of themes and to make suggestions as to the most “foundational” to the most “invested” forms of providing respect in our orientations to patients.

Two main themes emerged regarding respect as evidenced by medical professionalism: (1) physician availability and (2) considerations of privacy. These two themes are tied together because they are both specific to the delivery of healthcare, whereas the ‘orientation’ themes could be described in any interpersonal relationship.

### Respect in provider’s orientation towards patient

#### Treated as a person

Participants described being treated as a person as a fundamental component of respect from their physicians. This, in general, was defined as being more than a patient or a disease. Participants described this as being known and having their personal life inquired about. Examples included when physicians used patient’s name to greet them, looked at patients in the eye, asked patients how they were doing, inquired about non-medical aspects (e.g., about work, family, friends). One participant stated,

“*I think it is important that the doctor always knows your name and that he looks at you and he greets you with your name. Because they see many patients and the fact that your doctor remembers your name, that makes me feel that they are taking me into account and that I am respected*” (41 years old, White, Hispanic, from Nicaragua).

When asked about how they know their doctor respects them, another participant said, “*They ask you about your everyday life. They don’t just come in and ask you about the regular stuff and write it*” (56 years old, Black, Non-Hispanic, from Jamaica). This participant described how the doctor was not just interested in the physical health of the patient, but was also interested in the person, beyond the disease and disease management issues at hand. Friendly greetings were also described as a sign of respect and an acknowledgement of being seen as a person, “*Every time I bump into him, he says, ‘Hello, I’m glad to see you, I’m glad you’re here’. A couple days ago I was in the building and I saw him and he stopped he took a moment he said, ‘How are you doing?*’ (44 years old, White, Hispanic, from the US). One participant described a negative case example,

"*Some people won’t look you in the eye if you are talking to them or they turn away doing something else or they’ll pay little attention to the things you say, as if you were unimportant. That’s not right, that’s not treating someone like a person. But if you are talking to them and they look you in the eye and you can tell that they are interested in what it is you have to say, that means the person respects you, and considers you a person*" (46 years old, Black, Haitian, from Haiti).

Taking the time to greet and look at the patient in the eye was a demonstration of being treated as a person. Patients connected these behaviors to being worthy of the time and attention of the provider.

#### Treated as an equal

Another major foundational theme centered around being treated as an equal as a sign of respect. This was defined as being collaborators (i.e., physician and patient) in care and treatment decisions. Being treated as an equal also meant encouraging the patient to participate in conversation. Illustrative examples included instances when physicians encouraged patients to talk about symptoms and care plans, encouraged patients to ask questions, provided opportunities for both listening/explaining, and demonstrated overall partnership with patient (e.g., being “*in this together*”). For example, one participant explained:

“*She makes it easier, easy for me to talk about my health condition and not be afraid of it. She talks to me with respect. She makes me feel loved, like she makes me feel a connection, like we got a bond, and this is something like, we in this together, ‘cause I am here to help you*” (38 years old, Black, Non-Hispanic, from the US).

This was as opposed to an example of disrespect when a participant described,

“*I guess the way she was talking to me, like when she first sees me it was like, ‘You going to do this, you’re going to do that.’ And I was like, ‘I understand everything you saying, but me and you both grown people. You talk to me in a respectful way. I talk to you in a respectful way.’ So we squashed that*” (58 years old, Black, Non-Hispanic, from the US.

Another aspect of being treated like an equal was taking the time to explain procedures and results to patient. The participant described when their doctor*, ‘go through whatever* —*my blood work and stuff. He explains it to me, “This is OK. That’s OK.” You know, as far as the blood work, every time I come, he [explains that] everything is still low: cholesterol, my sugar. Everything is fine*” (51 years old, Black, Non-Hispanic, from the US.

#### Treated without blame or prejudice

Being treated without blame or prejudice emerged as another prominent theme in participant’s description of what constitutes respect from providers. This was defined as avoiding putting shame or guilt on the patient and acting without prejudice or stigma towards the patient. Examples included standing up for the patient in interactions with other family members and with other providers, avoiding talking down to the patient, avoiding placing blame on the patient, and treating the patient without prejudice. A negative case example included,

“*She talked down at me, number one. Number two, you make me feel like I'm dirty. You know, like I did something wrong, you know, instead of rubbing my hand, and saying, ‘It's going to be OK. We alright. You know, you alright.’ You're looking down and you really looking down your nose sideways at a person, you knows... She is there for the money. Put it like that. Not for the comfort, like the bedside manner the doctor's supposed to have*" (64 years old, Black, Non-Hispanic, from Honduras).

The participant described how the provider acted towards her with disapproval and made her feel like she was dirty, an experience that may be particularly re-stigmatizing for WLH who are already likely to experience discrimination based on the stigma associated with HIV. The participant then gave a concrete example of a small, yet actionable example of what the patient would have wanted instead: a physical touch, reassurance from the doctor that they are going to be “ok”, and comfort. For another participant, being treated with respect meant without prejudice, which was described as,

“*Well for me, it’s like to be told something that I don’t like, like a question about the disease or a rejection. I don’t know for other people but she grabs me gives me a hug, gives me a kiss, whether I have the thing or not*” (63 years old, White, Hispanic, from Venezuela).

#### Treated with concern/emotional support

Finally, being treated with respect was also manifested through concern and emotional support from the provider. Examples included acknowledging, consoling, and comforting the patient in times of distress, providing warmth and love towards patients, expressing interest and concern for the patient, and providing reassurance. One participant described,

“*She comforts me, I cry to her. She give me a hug. And she try her best to do whatever she needs to do to keep my healthy*” (38 years old, Black, Non-Hispanic, from Jamaica).

Another participant describes her physician’s level of concern.

“*And then you come back to the doctor and you put on weight. And he will say to me, ‘[Name], your weight was this last couple times you were here. What’s going on with you?’ And he will not stop until I let him know. ‘Why are you stressed out?’ You know so it’s, it shows me that he care. It’s not only because I’m his patient. And you know, It’s not the dollar, it’s the individual person. That’s how I see it, you know*” (63 years old, Black, Non-Hispanic, from St. Thomas).

### Respect in medical professionalism

Respect manifested in two main areas related to medical professionalism: availability of the provider and privacy during medical visits. Availability included having timely access to the physician and allowing the patient to speak to and see the doctor without appointments. One participant described:

“*I had a question and it’s not like I had to make an appointment to ask him the question. I stood there with him, or he stood with me and we discussed it for a moment. And he asked me when my next appointment was. It was a week later and he’s like okay well do what you got to do… He doesn’t treat me like a number or a paycheck, he treats me like a person. Like we’re humans, like we’re people, we’re not a statistic*“ (44 years old, White, Hispanic, from the US).

Respect experienced through maintaining privacy encompassed both ensuring confidentiality during medical visits and asking for consent to have additional healthcare workers or students while in the room with the patient. Examples also included providing spaces and utilizing practices that allow for privacy in waiting rooms. One negative case example from a participant described, “*I always feel like I’m treated with respect when I go there. Except for when I tell them they got me sitting in this wide-open place with no privacy*” (58 years old, Black, Non-Hispanic, from The Bahamas). Another described a lack of respect when including additional care team members without patient consent. “*I do feel uncomfortable when there are people there, like the students. I wouldn’t mind if it was the nurse... But is disrespectful that they don’t ask you for permission at all. That it is*” (47 years old, White, Hispanic, from Honduras).

## Discussion

Results from this study confirm previously identified aspects regarding patient experiences of respect while also expand the understanding of respect to include additional aspects related to delivery of care and interpersonal relationships among a diverse group of WLH. Other studies have found the importance of being treated as a person and being treated as an equal as a demonstration of respect [10]. In our findings, being treated as an equal included instances when patients were encouraged to participate in their care, ask questions, and have a partnership in the decision-making process. This contrasts with commonly accepted practices of non-interference where the provider is solely the deliverer of health information. Other qualitative work with HIV patients has highlighted the importance of not simply giving information to the patient, but rather facilitating engagement and understanding with the patient [24]. Two additional aspects of respect identified among our sample included being treated without blame/prejudice and being treated with concern/emotional support. We suggest these are the more “invested” forms of showing respect because they may require more effort on the part of the physician in practice and because they seem to build on the prior two themes; that is, without being treated as a person or an equal, it is unlikely the provider would be able to treat the patient without blame or provide them with emotional support. These four themes, together, offer a more robust understanding of what respect for persons entails in patient/ provider interpersonal relationships than is currently available in the literature and point to directions of future research to improve respect as a part of biomedical ethics training.

Being treated without blame/prejudice and being treated with concern/emotional support may be particularly important and salient for our population of focus and other populations who experience compounded levels of stigma and discrimination, both internalized and externalized. Moreover, these themes are important in the context of considering the social and structural determinants of health that are known to impact disease progression and maintenance in care. Despite clear understanding of the impact of social and structural determinants on medical interventions and subsequent health disparities [25], providers are often inadequately prepared to assist patients in addressing these issues [26, 27].

Reducing blame of individuals who have largely experienced social and structural constraints that impact health could be a potential way that providers can make progress in providing better informed, patient-centered care that is aligned with the way patients experience respect. Showing concern and providing emotional support could be a way to counter negative psychosocial consequences that result from stigma and discrimination and that further exacerbate disparities in health care outcomes. Failing to reduce cycles of blame or failing to provide emotional support and concern may perpetuate structural violence, or harm that is inflicted on a patient due to structural realities and circumstances beyond their control [26]. For example, a participant reported that the doctor demonstrated respect when they expressed concern when her weight fluctuated, as opposed to getting upset with her or blaming her for not handling her life circumstances and stressors (i.e., stress from children) differently.

Women also described respect as a part of medical professionalism. While these two components of professionalism are not usually grouped in the realm of bioethics, results have implications for the organization and delivery of care. Medical practices should take into account how the availability of physicians and considerations of privacy impact their patients’ perceptions of being respected. Other related qualitative work regarding respect in healthcare found similar results. Bridges and colleagues found that organizational procedures for scheduling were integral to patient-perceived respect. Their findings highlight how institutional policies and procedures play a role in building respectful relationships, from the perspective of patients [14]. Results suggests that decisions of scheduling, time management, and teaching become issues tied into bioethics and are not simply procedural management decisions.

Our study is not without limitations. First, the participants in this study were all receiving services from the RWP and thus, have different experiences from those not enrolled. Also, while a strength of our study is the inclusion of women from diverse ethnic and racial backgrounds, we did not have enough data saturation to make comparisons across groups (e.g., Hispanic WLH versus Haitian WLH experiences of respect). The current analysis was conducted with the data set as a whole; after initial analysis, researchers looked at responses from sub-groups of women individually to see if there were unique patterns in responses when compared to other sub-groups. Researchers did not identify major distinctions between sub-groups of women and therefore present results collectively. We acknowledge potential differences in experiences of respect may exist, and it is possible that we did not have enough data in each subgroup to make these comparisons across groups. Finally, the findings presented in this paper come from 57 of 74 interviews that were conducted as a part of the parent study. Only 57 participants were explicitly asked a prompt question about experiences of respect and, thus, only their data was included in this analysis. Additional data regarding experiences of respect could further enhance the understanding of participant perspectives.

## Conclusions

Findings reflect that participants viewed respect more broadly than traditionally conceptualized in medical bioethics, and our findings offer a more robust understanding of how respect for persons can be actualized in practice. Findings have implications for broadening areas of focus of future bioethics education, training, and research to include components of interpersonal relationship development, communication, and clinic procedures. These areas of training may increase respectful medical care experiences and potentially serve to influence persistent and known social and structural determinants of health through provider interactions and health care delivery.

### Supplementary Information

**Supplementary Material 1.**

## Data Availability

The datasets used and/or analyzed during the current study are available from the corresponding author on reasonable request.
