# Supplementary material for: How patients experience respect in healthcare: findings from a qualitative study among multicultural women living with HIV
Source: BMC Med Ethics. 2024 Mar 27;25:39. doi: 10.1186/s12910-024-01015-1 (PMC10967177; doi:10.1186/s12910-024-01015-1)
Supplement: Supplementary file 1 — Supplementary Material 1. [file 12910_2024_1015_MOESM1_ESM.docx]

Participant ID: __________

Language interview is being conducted in:__________________________________

Date: __________

IN-DEPTH INTERVIEW GUIDE FOR WOMEN

INTRODUCTION (begins after consent form is signed):

Thank you and purpose: **Thank you for agreeing to participate in this study. As you know, this study is about learning how the Ryan White Program can better address the needs of women living with HIV infection. We are specifically interested in knowing the things that help or prevent women with HIV from getting HIV care and seeing their HIV doctor regularly. Our goal is to learn how the Ryan White Program can serve women better.**

Confidentiality: **We will summarize the answers of all the women participating in this study to guide the Ryan White Program. Your individual answers will not be shared with your HIV doctor or the Ryan White Program. I want to be sure that you are comfortable with this process, and that you understand you may stop me and this interview at any time. All the information you share will be completely confidential. To keep things confidential, we will remove all names that may come up during the interview.**

Audiotaping: **You gave your consent to be audiotaped. If you wish to explain something and not be taped, please let me know at that time and I will stop the recording.**

*INTERVIEWER: [Start recording]*

GETTING TO KNOW PERSON:

**Before we start the detailed questions, I would like to ask a few questions about your background to get to know you.**

*INTERVIEWER: [Fill out separate background form now.]*

1. **Where were you born?**

- - Colombia
  - Cuba
  - Dominican Republic
  - Guatemala
  - Haiti
  - Honduras
  - Mexico
  - Nicaragua
  - Puerto Rico
  - United States (not Puerto Rico)
  - Other, please specify: ___________________________

2. **How long have you been living in the Miami area?**: ______________________

3. **What is your marital status?**

- Single
- Married/living together with partner
- Separated/divorced
- Widowed
- Other_______________________________

4. **How many children do you care for at home?**

- None
- One
- Two
- Three
- Four or more

5. If one or more, **please tell me the ages of the children:**____________________________

6. **Do you care for any older adults such as a parent or grandparent?**

- Yes, **please specify relationship**:___________________________________
- No

7. **How many years of school have you completed?**___________________________

8. [IF 12 OR MORE YEARS], **please tell me any degrees from high school, college or another school that you have?** [CHECK ALL THAT APPLY]

- - High school or GED
  - Vocational (trade school)
  - Associate’s degree in college
  - Bachelor's degree in college or higher (For example: BA, BS)
  - Other: _______

9.. **Are you currently working outside the home?**

- Yes
- No

10. [IF YES], **about how many hours do you work per week?** _________________

11. [IF YES], **what is your job?**____________________________

**Thank you for telling me about yourself. Now we are going to get into the detailed questions about your health care experiences. We will start by asking about your experiences with case managers and then with the doctors and nurses at the place where you get your HIV treatments. We are interested in your honest opinions and experiences. In answering the questions, we would like you to provide as many details as you can to help us understand your experiences. Please remember that there is no right or wrong answer to any of these questions, and your individual answers will not be shared with your doctors or case managers**.

Case manager

**Now, I’d like you to think about your current medical case manager and any other case managers you have had over the last 2 years.**

**1. Please describe your relationship with your current case manager(s)** *[use specific names if the participant has named them] [wait for participant to answer].*

*[Areas for probing: For each case manager mentioned, determine how long they have had this case manager. Determine the quality of the relationship (Helpfulness, satisfaction, etc.). Determine participants’ perceptions of being listened to or encouraged to express their needs. When appropriate, encourage participant to provide examples to illustrate qualities mentioned. Determine examples of things case managers do to make participant comfortable. If participant cannot provide examples of what the case manager does to make them feel comfortable, ask what specific things they wish the case manager would do to help them feel comfortable.*

**2. Some women prefer to have only women as case managers and prefer to have case managers who are of the same ethnicity. Other women do not have a preference about the gender or ethnicity of their case managers. In what ways does ethnicity and gender matter to your relationship with your case manager. Let’s start with gender.** *[wait for participant to answer].* **Now let’s talk about ethnicity**.

*[Areas for probing: Determine if similarities and differences in gender or ethnicity facilitate communication about specific issues (e.g. relationships, sexual relationships, asking for help) or hinder communication.]*

**3. Women living with HIV have unique needs and face unique challenges. Please tell me in what ways your case manager understands and is able to help you face the challenges that you have as a woman living with HIV?** *[wait for participant to answer].*

*[Areas for probing: Determine specific examples of how their case manager supports them. Are there things the case management program can do to do a better job of helping women living with HIV?]*

**Now, I’d like to talk about how your case manager communicates with you about your HIV care.**

**4a. Please describe what your case manager does to help you stay in treatment.**

*[Areas of probing: Determine what are some things that the case manager could do to help participant come to appointments].*

**4b*.* Describe what your case manager does to help you take your medication as prescribed.**

*[Areas of probing: Determine specific things that the case manager does to help participants take HIV medications as prescribed.* **How does your relationship with your case manager motivate you to take your HIV medications as prescribed?]**

**5. During the last 2 years, have you needed to be referred to a service such as dental care, substance use treatment or legal services?** *[NOTE: If no, please go to question 6.] [ If yes,]* **please describe the experience with the referral process.**

*[Areas of probing: Determine specific services referred to. Determine perception of satisfaction of referral process. Provide specific examples of a referral process to one of these services.]*

**Now we will talk about the agency where you receive your case management services.**

**6. Please describe** **any experience you may have had in providing feedback to the agency where you get case management about services it offers and the program staff.** *[wait for participant to answer]*.

[*Areas of probing: Determine awareness of the mechanisms to provide feedback (e.g. surveys or advisory boards). If participant has not had such personal experiences, inquire if the participant is aware of other women who have provided such input and under what circumstances. If you had a chance to provide input, what would you want to say?]*

**7. What would be good ways for the case management program to get input from women living with HIV?** *[wait for participant to answer]*.

*[Areas of probing: Determine ways the program can best allow women to share sensitive and important information that could help the program make changes. Elicit specific examples or recommendations.]*

Health care providers

**Now I would like to ask you some questions about your experiences with the doctors and nurses, where you are prescribed your HIV medications.**

**1. I would like you to think about the HIV doctor or doctors who have cared for you during the last two years. Please describe your relationship with them.** *[wait for participant to answer].*

*[Areas for probing: For each doctor mentioned, determine how long they have had this doctor. Determine the quality of the relationship (Helpfulness, satisfaction, etc.) When appropriate, encourage participant to provide examples to illustrate qualities mentioned.] Determine how participants have been treated by doctors and nurses. Ask participants to provide examples of instances when they were treated with respect, and instances when they were not treated with respect.*

*[NOTE: If participant says that they have a nurse practitioner as their primary HIV care provider, please use nurse practitioner instead of doctor in the questions below.]*

**2. Thinking about your current HIV doctor, what are some things your doctor does to get to** **know you as a person?** *[wait for participant to answer].*

*[Areas for probing: Determine how well doctors remember things about them. Determine how doctors show interest. Ask participants to provide examples of when their doctor tried to get to know them as a person and their family. What would you like them to say and do to show they care for you as a person?]*

**3. Describe the ways that your HIV doctor involves you in decisions about your treatments?** *[wait for participant to answer].*

*[Areas for probing: Determine participant’s level of involvement regarding medication, tests, non-HIV health issues, etc. Ask participants for examples of when their doctor involved them in their treatment.]*

**4*.* Please provide examples of things that your doctor and nurses do that help you feel comfortable and welcomed during your appointments?**

[*Areas for probing:* *If participant cannot provide examples of what the doctors and nurses do to make them feel comfortable during their appointments, ask what specific things they wish the doctors and nurses would do to help them feel comfortable. Determine if there are specific things that make the participant feel uncomfortable.]*

**5. Please tell me what your HIV doctor and nurse do to help you stay in treatment for your HIV.**

*[Areas of probing: Elicit examples of things that make it difficult to keep appointments. How the doctor and nurse have helped.]*

**6. Please tell me what your HIV doctor and nurse do to help you take your HIV medication as prescribed.**

*[Areas of probing: Determine participants’ perceptions of having medications explained to them. Encourage participant to provide examples of what the doctor does to help them understand things related to their HIV medication (e.g., Clarity, relevance) Elicit examples of things that make it difficult for the participant to take HIV medications as prescribed and how the doctor or nurse have or have not helped.* **How does your relationship with your doctor or nurse motivate you to take your HIV medications as prescribed?***]*

**7. Some women prefer to have only women doctors, and some women prefer to have doctors who have the same race or ethnicity as they are. Other women do not care. In what ways does ethnicity and gender matter to your relationship with your doctors? Let’s start with gender.**  *[wait for participant to answer].*  **Now let’s talk about ethnicity.**

*[Areas for probing: Determine if similarities and differences in gender or ethnicity facilitate communication about specific issues (e.g. relationships, sexual relationships, asking for help) or hinder communication.]*

**8. During the last 2 years, have you needed to be referred to a medical specialist?** *[NOTE: If no, go to question 9.] [If yes],* **Please tell us what that referral process was like.**

*[Areas of probing: Determine specific specialists referred to. Determine perception of satisfaction of referral process. Provide specific examples of how a referral process to one of these specialists goes.]*

**9. We have talked about your relationship with your doctor, nurse, and case managers. However, we know that other support staff such as front desk, laboratory, peer support, and transportation staff are also important. Please tell me your overall experience with each of these support staff.**

[*Areas for probing: Determine how certain staff or services are helpful or not helpful in their HIV treatment. Provide a specific example of how support staff are helpful or not helpful].*

**Is your HIV doctor in the same location as your case manager?**  *[NOTE: If no, go to question 10. If yes, go to question 11]*

**10. Please describe any experience you may have had in providing feedback to the agency where you get primary healthcare about services it offers and the program staff.** *[wait for participant to answer].*

*[Areas of probing: Determine awareness of the mechanisms to provide feedback (e.g. surveys or advisory boards). If participant has not had such personal experiences, inquire if the participant is aware of other women who have provided such input and under what circumstances.]*

**11. One last question, please describe ways your clinic, agency, case manager, doctor and nurse work to help women when they are afraid to tell their family members that they are infected with the HIV virus.**

[*Areas for probing: Determine if participant or other women that they know have sought help with disclosure. Ask participant how the healthcare providers can improve in this area.]*

CONCLUSION: **Thank you so much for your time. We are almost done, but before we finish, is there anything else you would like to add or share at this time?** *[wait for participant to answer].*

**THANK YOU AGAIN.**
